# The support and information needs of adolescents and young adults with cancer when active treatment ends

**DOI:** 10.1186/s12885-020-07197-2

**Published:** 2020-07-28

**Authors:** Sarah Lea, Ana Martins, Lorna A. Fern, Matthew Bassett, Maria Cable, Gary Doig, Sue Morgan, Louise Soanes, Michael Whelan, Rachel M. Taylor

**Affiliations:** 1 Cancer Division, University College London Hospitals NHS Foundation Trust, London, UK; 2 Teenage Cancer Trust, London, UK; 3 Coventry University, Coventry, UK; 4 Leeds Teaching Hospitals NHS Trust, Leeds, England; 5 Centre for Nurse, Midwife and Allied Health Profession Led Research (CNMAR), University College London Hospitals NHS Foundation Trust, 1st Floor East, 250 Euston Road, London, NW1 2PG England

**Keywords:** Young people, Teenagers, Adolescents, Young adults, Cancer, Healthcare transition, Early survivorship, End of treatment

## Abstract

**Background:**

The end of active treatment is a period of high stress for young people with cancer, but limited literature exists about their information and support needs during this phase. This study aimed to understand the needs of young people with cancer, how these needs are currently being met, and how best to provide information and support at the end of active treatment.

**Methods:**

This was a multi-stage, mixed methods study exploring the end of treatment experience from the perspectives of young people, and the healthcare professionals caring for them. Semi-structured interviews were undertaken with healthcare professionals, which informed a survey administered nationally. Subsequently, semi-structured interviews were conducted with young people. These combined results informed a co-design workshop to develop recommendations.

**Results:**

Telephone interviews were conducted with 12 healthcare professionals and 49 completed the online survey. A total of 11 young people aged 19–26 years (female = 8; 73%) were interviewed. The stakeholder workshop was attended by both healthcare professionals (*n* = 8) and young people (*n* = 3). At the end of treatment young people experience numerous ongoing physical issues including pain, fatigue and insomnia; in addition to a range of psychosocial and emotional issues including anxiety, fear of recurrence and isolation. The top three priorities for end of treatment care were: earlier provision and preparation around on-going impact of cancer and cancer treatment; standardised and continued follow-up of young people’s emotional well-being; and development of more information and resources specific to young people.

**Conclusion:**

The access and availability of appropriate information and sources of support at the end of treatment is variable and inequitable. Young people’s needs would be more effectively met by timely, structured and accessible information, and support provision at the end of treatment to both prepare and enable adaptation across their transition to living with and beyond cancer. This will require both organisational and practical adjustments in care delivery, in addition to a renewed and updated understanding of what the ‘end of treatment’ transition process means.

## Background

A cancer diagnosis in adolescents and young adults (AYA) presents unique challenges with diagnostic pathways being generally protracted and complex, along with inequitable access to care and research. Subsequently the end of treatment is often accompanied by fear and uncertainty [1–5]. Young people feel unprepared for life after treatment and experience a new focus of concern around their wellness, health maintenance and future [5, 6]. While there has been much written about the provision of long-term follow-up care, and models proposed to address these needs [7–11], there has been less focus on the point where active treatment ends. Guidance in England proposed young people should be reviewed by the multi-disciplinary team (MDT) prior to entering survivorship [12] and should ‘*be provided with access to resources and/or referral information that can help them re-integrate back into “normal” society*’ [6], p.210. While these recommendations are useful, more specific guidance would provide professionals with a clearer direction of how they can provide support for young people at this point in the cancer timeline. The limited evidence which currently exists suggests young people leave active treatment with little support and experience an unanticipated withdrawal of services [13].

We have previously shown that the end of treatment represents a period of stress for young people. This includes: the challenges of social reintegration and finding their self-identify; the expectation versus the reality of treatment ending; and the sudden loss of the safe ‘bubble’ of treatment [14]. However, little is known about young people’s information and support needs specifically at this time point, and to what degree these needs are met. This paper presents study findings related to the following aims:

1. To identify young people with cancer’s information and support needs when active treatment ends
2. To determine whether a current model of care fulfils young peoples’ information and support needs
3. To use co-design with stakeholders to draft recommendations based on the results of the study

## Study design

This was a multi-stage, mixed methods study conducted from January to December 2018. This study was approved by London - South East Research Ethics Committee (Reference: 15/LO/0299) and the Health Research Authority (Reference: 236864). The methods of data collection and findings from healthcare professionals and young people are presented sequentially. These informed the co-design workshop to develop recommendations.

## Patient and public involvement

Involving patients and key stakeholders, i.e., professionals, in study concept and design is essential in research [16]. As such, prior to commencing this study we ran two workshops, one with young people and a second with AYA nurses, the main professional group providing end of treatment care for young people in the United Kingdom (UK). The aim of the workshop with young people was to ascertain the current experience of end of treatment to inform the healthcare professional interview schedule and to determine study acceptability to young people. The one-day workshop included our existing Young Advisory Panel (YAP), a group of young people with a previous cancer diagnosis, who were experienced at assisting the research team with study design and content [17]. The YAP assisted in study design, interview content and study recommendations. We invited YAP members onto the research group, but their preference was to attend the final workshop as stakeholders.

The aim of the workshop with nurses was to determine from the point of professionals what young people’s needs were at the end of treatment and up to 1 year post treatment; how these were currently met; whether their own service meets these needs and what additional support, information or services would benefit young people at the end of treatment. This information was used to inform the interview schedule for the interviews with healthcare professionals. Two team members (LAF/RMT) ran the workshop at an existing one-day event held by the study funders for nurses.

## Methods

### Healthcare professionals

#### Objectives

- To describe the models of providing information and support currently provided in the UK when active treatment ends using semi-structured interviews.
- To quantify the frequency of models of information and support using an online survey.

#### Sample and setting

A purposive sample of healthcare professionals working in specialist AYA treatment centres, as well as other cancer settings where young people received care, from across the UK were invited to participate in a telephone interview. This was to represent different professionals, geography and healthcare settings. Verbal consent was recorded at the beginning of the interview.

#### Data collection

##### Interviews

Healthcare professionals were invited to participate in a telephone interview, which explored how they were currently supporting young people at the end of treatment and what additional information and support they thought young people needed. A semi-structure interview schedule guided the telephone interviews, which was developed from the literature review [13] and the patient and public involvement activities described above. This was not prescriptive and was purposefully flexible to enable the researcher to explore models of end of treatment care in different settings, and to allow the views of the healthcare professionals who took part to also shape the direction of the discussion.

##### Survey

A healthcare professional survey was developed to build on key themes from the telephone interviews. The survey comprised of closed-ended questions to determine the extent to which end of treatment-specific care was available, healthcare professionals’ perceived need for end of treatment-specific care, and whether there were variations in the availability of end of treatment-specific care (i.e. geographic or hospital-type inequalities). The survey also included a free text comments box to give participants the opportunity to add additional opinions about the provision of care at the end of treatment. The content of the survey was confirmed through review by professionals working in AYA cancer care and experts in survey design. The link to the online survey and a word version was distributed via email through TYAC membership (*n* = 437; the organisation representing professionals working in AYA cancer care in the UK), Teenage Cancer Trust funded staff (*n* = 56) and through healthcare professional contacts working in four study sites. The link to the online survey was shared online via Twitter. Completion of the survey was regarded as consent to participate.

#### Analysis

Interview data were digitally recorded, transcribed verbatim and analysed using Framework Analysis [18]. Key themes were identified which informed the development of the framework. Data were explored in more depth through a series of structured steps: becoming familiar with the transcripts, indexing the transcripts according to the framework, charting data from the transcripts then as a team the charts were reviewed and interpreted. Quantitative survey data were analysed descriptively. The interview and survey data were analysed separately but synthesised with interview data at the point of interpretation to inform the co-design workshop.

### Young people

#### Objective

To identify young people’s information and support needs when treatment ends and to determine whether there is a current model of care that fulfils young people’s information and support needs during this period of their cancer timeline.

#### Sample and setting

Details of the methods for data collection from young people have been reported previously [14]. In summary, we aimed to recruit 30 young people whose treatment had recently ended and whose treatment ended approximately 12 months previously. They were eligible to participate if they were aged 16–29 years at the time of the study. Young people were recruited by the local clinical members of the AYA MDT in four participating sites. The AYA MDT in the participating sites were used to identify young people in both the specialist and non-specialist AYA cancer units to ensure the experiences of young people with little/no specialist care were represented. Young people were also invited to take part in the study through adverts on Teenage Cancer Trust social media and through their mailing list of young people who had consented to receive information about non-fundraising projects. Young people could only participate when the research team had received a copy of the signed consent form.

#### Data collection

Data were collected using multiple methods to facilitate inclusion, taking into consideration disability and geographical location. Young people had the option to participate in a telephone or face-to-face interview. The telephone and face-to-face interviews followed a semi-structured schedule developed through the review of the literature [13] and YAP workshop. This was not prescriptive and was flexible to enable the researcher to explore new and emerging experiences. Interviews were held at a time (and location for face-to-face interviews) of the young person’s choice.

#### Analysis

Data were digitally recorded, transcribed verbatim and analysed using Framework Analysis [18] in the same way as analysis of healthcare professional interviews.

## Results

### Healthcare professionals

Interviews were conducted with 12 healthcare professionals (nurses *n* = 8; youth support coordinators (YSC) *n* = 2; medical doctor *n* = 1; psychologist *n* = 1) and 49 healthcare professionals completed the online survey. Most survey participants were nurses (*n* = 30; 61%), who worked in a specialist AYA cancer service, or in both the specialist service and other adult cancer services (*n* = 40; 82%). Most survey respondents had worked with young people with cancer for more than 3 years (*n* = 39; 80%). Participants represented a range of geographical locations, with coverage across all four UK countries (Table 1).

**Table 1:** Characteristics of professionals completing the online survey

Characteristic	n (%)
*Total*	49 (100)
*Role*	
Nurse	30 (61)
Youth support coordinator	9 (18)
Medical doctor	4 (8)
Other	6 (12)
*Place of work*	
AYA specialist hospital	26 (53)
Across both an AYA specialist hospital and Non AYA specialist but provider of AYA care	14 (29)
Non AYA specialist but provider of AYA care	5 (10)
Other	4 (8)
*Time working with young people with cancer*	
< 1 year	4 (8)
1–3 years	6 (12)
> 3 years	39 (80)

*AYA* Adolescent and young adult

The interviews with healthcare professionals identified five overarching themes: the issues young people experienced at the end of treatment; the mechanisms by which these were identified; the facilitators and challenges for identifying young people’s needs; the existing sources of support and information available to young people; and the perceived changes that were needed to improve care for young people at the end of treatment. The survey, which was developed from these interview data, explored these themes further. Survey data corresponding to each theme are presented in addition to the interview data, to provide extra detail to the findings.

#### Issues experienced by young people at the end of treatment

Healthcare professionals perceived young people to have a range of issues at the end of treatment, which fell into the following categories: physical, mental health, emotional, spiritual, social/relationship and education/employment issues. Issues within all of these categories required young people to adapt in some way and needed to be addressed in order for them to establish a ‘new normal’. Healthcare professionals recognised that young people required advice and support to navigate these various aspects of their life in the months after completing cancer treatment:

*“Last week I had a patient who’s probably two months post-transplant, and just rang me up and asked me for advice on how to get back into work because apparently, her work were being quite tricky about doing a phased return to work, and she asked for advice.”*

**Fig. 1:**
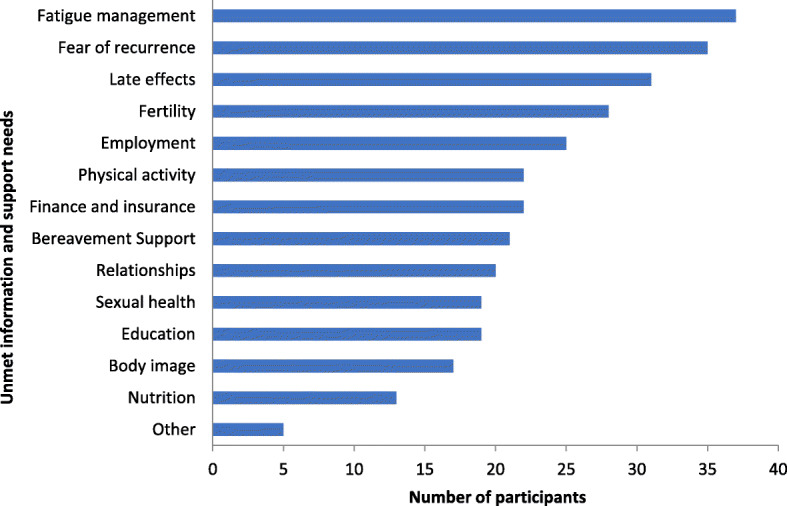
The main unmet information and support needs at the end of treatment (*n* = 49)

In terms of provision of information and support that met these issues, healthcare professionals perceived young people to have a variety of unmet needs. They felt that fatigue management, fear of recurrence, late effects, fertility and employment were some of the key issues where young people’s information and support needs were unmet (Fig. 1).

Furthermore, in the interviews, healthcare professionals referred to ‘family needs’, recognising that young people’s parents, partners, and siblings may have questions, which may require referral to sources of information and support:

*“Then, also, a lot of partners sometimes ring me up and just-, things like asking advice on things like sex and relationships post-chemo, and again, it’s about signposting them to the right things.”*

#### How end of treatment issues were identified

Young people’s issues and needs at the end of treatment were identified in two main ways. The first was identification by healthcare professionals, either informally through mechanisms such as text messages initiating a conversation about issues that had arisen, or formally in a follow-up clinic:

*“The consultant had emailed me and said, ‘Just seen so-and-so, just to let you know these are their concerns. Have you got any ideas of how we can support?’ So, they might see them in clinic in a more formal setting, but over emails we’ll be linking up together.”*

Alternatively, needs were highlighted to healthcare professionals by young people themselves initiating a conversation about the issues they were experiencing; again, this was either formally or informally. In some cases, young people contacted healthcare professionals formally, making an appointment with a specific healthcare professional to discuss their issues, i.e. psychologist. In other cases, young people initiated an informal conversation through social media or text message:

*“It’s usually through text or through the Facebook group. That’s for young people who don’t come to the unit.”*

The survey asked healthcare professional’s assessment and identification of young people’s needs at the end of treatment. A formal or standardised tool for assessing the needs of young people at the end of their cancer treatment was used by 29 (59%) survey respondents, these most commonly included: the AYA ‘IAM’ (Integrated Assessment Mapping; *n* = 10; 20%); localised holistic needs assessment (HNA) (*n* = 8; 16%); Macmillan (UK cancer charity) e-HNA (*n* = 3; 6%); and other specified assessment tool (*n* = 8; 16%).

In terms of healthcare professionals specifically following up young people’s emotional and psychosocial needs at the end of treatment, 28 (57%) respondents stated that they used a formal or standardised process for this. Most professionals (*n* = 34; 69%) reported providing young people with specific information about their emotional and psychosocial needs at the end of treatment, and this was delivered in a multitude of formats, most commonly verbally (*n* = 30; 61%), but also online resources (*n* = 19; 39%), charity leaflets (*n* = 14; 29%) and local leaflets (*n* = 11; 22%).

Young person-initiated follow-up of issues and support seeking occurred through re-engagement with services for additional or specific support predominantly through directly contacting healthcare professionals, or someone in the service (*n* = 43; 88%). Alternatively, some healthcare professionals arranged to meet a young person alongside a clinic appointment (*n* = 31; 63%), and others received referrals from other healthcare professionals that a young person required support and needed to be contacted (*n* = 29; 59%). Additionally, survey respondents (*n* = 26; 53%) said that family members contacting them, or the service was another way that young people re-engaged with the service at the end of treatment.

#### Facilitators to provision of support and information

A variety of processes, services and roles were described by healthcare professionals to either facilitate or hinder the provision of information and support to young people. Processes were described such as specific meetings about the end of treatment that were used by the MDT, or end of treatment-specific services that were offered, for example, dedicated end of treatment nurse-led clinics. Facilitating roles included specific support from YSC, social workers and clinical nurse specialists (CNS), following treatment finishing:

*“Yes, they can ring us, they have our number, they are always welcome to ring us. We don’t ever really stop being a CNS, we have quite a good service and we carry a mobile and they can contact us.”*

The majority (*n* = 47; 96%) of healthcare professionals reported in the survey that young people were provided with contact details of a professional as an ongoing source of information and support after treatment ended. Similarly, 46 (94%) stated that their service offered one-to-one emotional, psychosocial and practical support for young people. Healthcare professionals ranked the specific healthcare professional roles which were the main point of contact, and the roles that were the primary sources of emotional and psychosocial support. The top three of each are listed in Table 2.

**Table 2:** Healthcare professionals’ views of the main point of contact for young people and the main source of 1:1 emotional psychosocial and practical support following the end of treatment

Following the end of treatment...^**a**^	
**The main point of contact for young people:**	**The main source of 1:1 emotional, psychosocial, and practical support**:
1. AYA Clinical Nurse Specialist (*n* = 36; 74%)	1. AYA Clinical Nurse Specialist (*n* = 38; 78%)
2. Youth Support Co-ordinator (*n* = 22; 45%)	2. CLIC Sargent Social worker/Community worker (*n* = 27; 55%)
3. CLIC Sargent Social worker/Community worker (*n* = 19; 39%)	
	3. Youth Support Co-ordinator (*n* = 25; 51%)

^a^multi-response question so numbers do not add to 100%; *AYA* Adolescent and Young Adult; CLIC Sargent is a UK wide cancer charity providing support for young people with cancer; Youth Support Co-ordinators, Teenage Cancer Trust funded social support staff for young people during and after treatment

The survey also explored the ways in which teams of healthcare professionals communicated and liaised with each other regarding young people’s issues and support needs at the end of treatment. Most commonly, this was through the AYA MDT meeting (*n* = 37; 76%). However, other mechanisms of healthcare professional communication were described: sending end of treatment summaries to general practitioners (*n* = 14; 29%); caseload management meetings (*n* = 6; 12%); bespoke end of treatment meetings (*n* = 5; 10%); and other mechanisms (*n* = 7; 14%), including handovers via email or documented on electronic patient records. Five healthcare professionals reported there were no formal discussions or communication about young people’s end of treatment issues or needs.

#### Challenges to provision of support and information

Numerous challenges that were identified that hindered the provision of information and support at the end of treatment. Specific professionals identified challenges in leadership, where services lacked the provision of a dedicated individual to lead this aspect of their service:

*“As a hospital no one person is, kind of, leading on it and there isn’t a set structure ( … ) everyone’s doing their own, sort of, separate thing in site-specific teams, it’s a bit loose.”*

Process challenges included difficulties in knowing where young people had been discharged to, for example, if the young person had returned to university they might have then been in a new city and new hospital. Some participants noted that young people did not need support and they were hard to engage:

*“The psychology team do a beading day [type of narrative therapy], and I think that can be quite useful at the end of treatment, looking back over things. Again, you can offer it to them all, but not many of them take it up.”*

The limitations of services, in terms of resource and time, were highlighted as another challenge to the provision of support at the end of treatment.

Healthcare professionals were asked to rank the challenges which emerged in the interviews that made it difficult to provide support and information to young people. The three biggest challenges were: lack of local services to refer young people to, i.e. mental health services (*n* = 18; 37%); geography/travel to services (*n* = 18; 37%); time constraints (*n* = 16; 33%). Additionally, healthcare professionals were asked what they felt the challenges were in engaging young people in existing charitable events/activities that were offered at the end of treatment. The key challenges were geography/travel to an event/activity (*n* = 39; 80%); young people being reluctant to attend an event unless they knew someone else who was going (*n* = 35; 71%), and that young people were restricted with time constraints, e.g. work (*n* = 34; 69%).

Specific groups were highlighted who were less likely to engage in support services offered at the end of treatment. These included: young men (especially young Asian men, independent men who lived greater distances from the hospital, and young men with testicular cancer); those who engaged less during treatment; those who lived rurally; younger teenagers; those with cognitive dysfunction; those with additional complex health needs; and young people who were socially deprived.

#### Existing sources of support and information

Existing sources were identified as being available to help young people. Healthcare professionals described the different hospital-based professionals and services young people were offered or could access, such as a psychologist. Other sources of support were from charities, local services and from peers either online or at social events. Sources of information included handouts, internet websites, and charity support days and groups. Healthcare professionals also suggested that young people obtained support through sharing advice and experiences with peers:

“Actually, I think the young people can find it quite reassuring to hear from other previous patients that, actually, it’s sh*t scary when you finish treatment because, the whole time during treatment, you’ve had these people looking out for you.”

Most healthcare professionals (*n* = 32; 65%) thought the service they worked in had the structures and processes in place to prepare young people for the end of treatment. However, 11 (23%) healthcare professionals felt there were inadequate structures and processes in place to prepare young people for the end of treatment in their service.

#### Timing of information and support provision

There were mixed views surrounding when information and support should be offered to young people in relation to their end of treatment. The difficulties of knowing when to provide information were reported, with issues sometimes not arising until young people were three to 6 months after treatment ended:

*“I think I tend to see more engagement with them maybe three to six months after treatment ends. That’s the time when they try to get back into normal life as much as they can but they, kind of, find that their friends have moved on or that things are different in work. They’re not quite as able to do the things they used to, so they, kind of, come back to us maybe three to six months on.”*

The survey results showed that most healthcare professionals (*n* = 36;74%) felt that the timing when information and support regarding end of treatment was provided was dependent on a young person’s circumstance. They felt the provision of both information and support should be given to young people at the following time points: 2–3 months before the end of treatment (information: *n* = 23; 47%; support: *n* = 18; 37%), directly at the end of treatment (information and support: *n* = 17; 35%), within the first month following treatment (information: *n* = 22; 45%, support: *n* = 19; 39%), or at 1 month after treatment ends (information: *n* = 11; 22%, support: *n* = 12; 25%).

#### Additional influencing factors: age, cancer type, personal situation

There were additional factors that needed to be considered, which influenced the whole process of young people accessing information and support at the end of treatment. These included their age, cancer type and personal situation.

*“It’s something we’ve probably got to look at. At the moment, they float in their own tumour groups … So, testicular cancer patients, 99% of them will be referred out, so, at their end-of-treatment, they will be at the [DH], so I don’t really get involved with them … Skin is a tricky one because a lot of skin, like melanoma, they don’t have an end-of-treatment because our link in with the treatment would be the nurse specialist for skin, but a lot of the time, they have it incised and that’s it.”*

As the healthcare professional interview data indicated that information and support needs were influenced by a young person’s cancer type, the survey explored whether needs were sufficiently met or unmet across a different cancer type. Both in terms of information and support needs, healthcare professionals identified certain cancer types to have more information and support available than others. Lymphoma, bone tumours and leukaemia were the cancer types with the most sufficient information and support available, and urinary, oral and lung cancer were considered the ones with less sufficient information and support available. It is essential to highlight that healthcare professionals also indicated that they did not know whether there was sufficient support available for many of the cancer types young people presented with.

#### Changes and improvements

There were several recommendations made by healthcare professionals to improve services at end of treatment. These recommendations fell into three categories: improvements related to services; roles; and processes. It was suggested that services should be reviewed to understand existing follow up processes, there should be provision of more information events and resources tailored specifically towards the needs of young people, such as end of treatment information evenings. Additionally, more services providing support for young people and to promote peer-to-peer support, such as social peer events for those who have finished treatment:

*“Young people supporting each other, I think, during the treatment, that’s one thing that certainly people benefit massively from, or they seem to anyway, is the connections they make with other young people. I guess, a lot of people continue that naturally, but how to maybe build that into finishing treatment, where they can still, there is still a way to maintain those connections through the hospital.”*

The importance of specific roles was discussed, such as the need for someone to lead and drive the development of end of treatment services. It was suggested that implementing more nurse specialist roles to support young people at this phase could be beneficial, in addition to an increased awareness of the significance of the end of treatment phase within young people’s cancer timeline, and their needs that surround this phase.

Healthcare professionals frequently spoke of the need to standardise and formalise follow-up processes at the end of treatment, and that this would improve equitability of services. A ‘graded approach’ to assist young people at end of treatment was suggested:

*“It’s that you have to have that balance between holding their hands for a bit longer, but eventually, they will let go and they’ll stay away, or you let them go straight away and then they’ll yo-yo back.”*

Suggested processes which could be formalised were the co-ordination of care, such as ensuring that follow-up clinic visits were not only medical in nature but were also combined with opportunities for young people to receive emotional or psychosocial support and information. Additionally, the need to standardise the holistic needs assessment process and the follow up of young people, to ensure consistency and equality in processes:

*“End of treatment is a huge part of that as well, so making sure that, whether we’ve been involved or not through treatment, that there is a contact at end of treatment and how that looks and what we offer.”*

### Young people

Eleven young people aged 19–26 years (females *n* = 8; 73%) participated in either a face-to-face or telephone semi-structured interview. Age at diagnosis was 17–25 years and participants either ended treatment < 6 months prior to interview (*n* = 7; 64%), or 6 months to 1 year prior to interview (*n* = 4; 36%). Eight young people were treated in specialist AYA services, two in adult services and one in both. Young people had a range of diagnoses, including carcinoma, leukaemia, lymphoma, bone tumours and germ cell tumours. Two had received a bone marrow transplant.

Similar to the healthcare professional data, there were several mechanisms by which young people’s issues were identified, and access to support was hindered by a number of challenges.

#### How issues were identified

Young people sometimes sought information in a formal clinic setting; however, these discussions mainly focused on their physical health needs. Less formally, support for their mental health, social and emotional needs was often sought through ‘bumping into’ or texting a professional they felt they could share their issues with. They expressed feeling like they needed to ask for support if they were having an issue related to their emotional well-being, as opposed to having anyone actively following-up this aspect of their well-being:

*“Normally, I have to ask for it, like you kind of have to tell them that you’re feeling like this, a bit. I feel like maybe a catch-up every now and again, maybe like a meeting. Well, I have clinic, but I feel like clinic’s very much doctor-orientated, whereas I think it would be more beneficial to be more, like, nurse-orientated.”*

There were young people who described feeling like they had all the information that they needed, but that was because they actively sought the support. While it was their responsibility to initiate a conversation if they felt they had any issues after treatment ended, some were regularly reminded of the help that was on offer to them should they need it:

#### Existing sources of support

The three types of professionals that young people reported getting the most support from were CNSs, YSCs, and social workers. They described having positive relationships with these professionals, and some continued their contact with them as a source of support during the first year after they finished treatment. Other sources of support that some young people found helpful was social media, including local hospital and charity Facebook groups. However, there were mixed views around using social media as a source of support. While some young people described finding it helpful as a way of meeting others or seeing positive or inspiring stories, others felt this was not helpful as seeing other ‘survivors’ doing really well after cancer treatment made them feel inadequate when they were struggling post-treatment. Additionally, online sources of information were described to be less helpful as they wanted information to be personal to them, with direct relevance to their questions and their situation.

Young people described finding face-to-face peer interactions a helpful source of support. Through meeting other young people and sharing experiences, it helped young people to process their cancer experience:

*“I think the social nights just helped me put things into perspective because I realised how, not easy, but yes, easy my treatment was compared to other people’s. I have a guy who was on the same treatment as me, and so we were in at the same time, so we were in always on the same days and stuff like that. Me and him still chat, which is nice. I think it’s just nice to see what people are getting up to since treatment.”*

Charity events and trips were recognised as providing young people with a way of rebuilding their confidence, providing a safe space for them to be themselves, without feeling ‘labelled’ as someone who had cancer.

#### Challenges

Young people described several challenges in getting the support and information they needed at the end of treatment. Some felt that needing and accessing support was not warranted because they had completed their treatment and were therefore ‘well’; believing that attention should be on those who were unwell:

*“I’m normal again, I’m not poorly anymore, so their attention should be on the poorly people but you kind of still want to be poorly, as awful as that sounds. Like, it’s nice to be in that little group of their number ones, that type of thing, whereas now I’m just a normal person who they used to know so all their attention’s on the poorly people, which it should be, and that’s an awful and selfish thing to say really but you kind of want to part of it again.”*

Another challenge that was highlighted was a lack of clarity on who young people should contact if they had questions or issues. Some were given a specific number to call and did not find this a challenge, but others reported having contact with a variety of professionals and that they were therefore unclear on who they should or could contact. This was especially the case for help with practical, life-related issues such as employment and finances.

Finally, young people described their struggle to take initiative when they had finished treatment. They started to have questions or issues arose a few months after finishing treatment, and while they recognised they could have done with having support, they did not take the initiative to contact anyone for help, and felt they needed prompting or support to do this:

*“It wasn’t until I was sent a letter by my GP, that then I thought, ‘Actually, I think I need some help, in terms of speaking to a therapist.’ So, then I made an appointment with another doctor, he was like, ‘Well, we don’t offer it at the practice, but here are a list of numbers that you can call’ … when you’re given so much information, you tend to just put things off, and not take the initiative to sort it out, but I think you’re so preoccupied with other things that you then just don’t make the effort to do it.”*

#### Influencing factors

There were factors that influenced and affected the whole process, including age, cancer type, personality, and life situation. Young people who had received a shorter, less intense cancer treatment described feeling that they had not had enough time to process the fact they had had cancer:

*“Then, for me, my treatment only lasted three months, so that’d be at the beginning, so-, just so you have time to get your head around it… I don’t feel like I’ve had it.”*

There were young people who recognised that asking for help did not come naturally to them and that it was not in their personality to discuss their feelings or seek support to deal with their emotions. This therefore influenced the way they accessed support after they finished treatment and their awareness of what was available to them:

## Developing recommendations

The single co-design workshop to develop recommendations from the results included young people, healthcare professionals and other key stakeholders, e.g. third sector representatives. Invitations to the workshop, along with detailed information about what it would entail were sent to young people who participated previously This was also given to young people by participating clinical teams, adverts on social media and an invitation was sent by Teenage Cancer Trust to a mailing list of young people who consented to receive information about non-fundraising projects. Healthcare professionals and key stakeholders were identified by members of the research team based on their knowledge of currently available services.

Prior to the workshop participants were sent the seven recommendations/priorities identified in the findings from healthcare professionals and young people (Table 3 and Additional file 1). These priorities represented a summary of the key issues they identified. Participants had the overarching theme/priority and a more detailed explanation of what was included in each priority. They were asked to rank them in order of importance with a brief description prior to attending the workshop. At the workshop participants were invited to share their top three and bottom three priorities and briefly explain why they had allocated them as top or bottom priority. This was recorded on a flipchart followed by a discussion of the ‘who, when, what and how’ for each of the top 3 recommendations.

**Table 3:** The end of treatment priorities identified from phases 2 and 3

1) Earlier provision and preparation around ongoing impact of cancer and cancer treatment	
2) Standardised and continued follow-up of young people’s emotional well-being	
3) Development of more information and resources specific to young people	
4) Increasing the availability and awareness of peer-to-peer support for all young people after treatment ends	
5) Increasing awareness of support available to young people at the end of treatment	
6) Clearer structures, roles and processes in place to assist young people to access support after treatment ends (e.g. definition of who is responsible for giving information and support at the end of treatment and how this is shared with the young person)	
7) Improved communication and care co-ordination between all professionals involved in a young person’s care after treatment ends	

### Study recommendations

Eleven participants attended the stakeholder workshop, which included young people (*n* = 3) and healthcare professionals (*n* = 8), representing the range of healthcare professionals that young people had reported providing most support: social workers (*n* = 2), YSC (*n* = 4) and nurses (*n* = 2). In all phases of the study, there were healthcare professionals and young people from different regions in the UK.

In the final stakeholder workshop, there was consensus on the top three priorities identified by the professionals for improving support and information for young people at the end of treatment. However, the group were split on their opinions on peer-to-peer support and therefore this was also explored further. The final top 3 priorities to improve end of treatment support for young people were identified as:

1. Earlier provision and preparation around on-going impact of cancer and cancer treatment
2. Standardised and continued follow-up of young people’s emotional well-being
3. Development of more information and resources specific to young people

The group then went on to decide ‘who’ was best placed to provide each of the priorities, when they were best delivered and in what format (see Supplementary file 2).

Due to the mixed views on the priority ranking of *‘increasing the availability and awareness of peer-to-peer support for all young people after treatment ends’* further discussion explored the group divide. There was consensus that peer-to-peer support was important and helpful for young people, however some healthcare professionals felt that this was already being offered and executed effectively. The benefits of online peer-to-peer support were discussed. While young people wanted to use social media as a way of communicating with other young people, they expressed that a peer to peer app would be more useful to them if it was part of an ‘app’ or platform that they were already using.

Young people described how attending charity events provided valuable opportunities to meet and connect with other young people. This was particularly important for those who had been cared for in adult services and had therefore not met other young people when they were on treatment in hospital. Young people reflected on how these people had impacted their life as a young person beyond their cancer experience:

*“She’s gone from being my cancer friend to my friend, and I think that’s part of the peer support for after treatment, is showing people that it’s not just necessarily dumping all your cancer friends and then getting ‘life after’ friends. You can bring those people with you and they go from being your cancer friends to your friend. Yes you can talk about the cancer stuff, but you can also talk to them about just the general life stuff as well.”* (Young person)

It was recognised in the workshop that peer-to-peer support was an essential part of assisting young people to transition from the end of treatment and to assist them to adapt to ‘normal’ life as a young person.

## Discussion

Our study showed there are many contributing factors, steps within the process, and challenges associated with delivering information and support to young people as they ended their cancer treatment, described by healthcare professionals and young people. Healthcare professionals identified several categories of issues that young people faced at the end of treatment. These issues were either identified by healthcare professionals, or highlighted to them by young people, and this was either formally in clinic or informally through text/social media. Formalised or standardised assessment tools to assess young people’s issues were used, such as the IAM online portal (https://tyaiam.co.uk), but not in all services. Consistent, formal, and mandated use of such tools to assess young people’s needs at the end of treatment was suggested as a change that could improve equitability of issue identification and therefore enable timely provision of support to all young people.

Our synthesis of the healthcare professional and young person perspective led to seven recommendations. To facilitate the development of future interventions, the co-design workshop aimed to rank the key priorities for changes and improvements to end of treatment services and processes for young people. It was felt that earlier provision and preparation around ongoing impact of cancer and cancer treatment was the most important recommendation because if this was done well, the process of supporting young people when treatment ended would be easier as they would be better prepared. Information should be given gradually throughout treatment by the healthcare professional who had a relationship with them, the ‘best-placed person’.

It was agreed that the ‘best-placed person’ should take responsibility for continued follow-up of young people’s emotional well-being after they finish treatment. It should be mandated that all young people have an HNA and were discussed formally by the MDT at the end of their active treatment. Using a framework to guide and track their contact with young people would assist them to follow-up their emotional well-being in a structured way. While it was recognised that this should be patient-led, every young person should be contacted within 1 month of treatment ending.

Development of more information and resources specific to young people was a priority to improve the provision of information. Healthcare professionals required more tailored, quality-assured and age-appropriate resources to provide young people with information particularly around fatigue, fear of recurrence, exercise and returning to physical activity, fertility, and other topics related to transitioning into adulthood and being a young person. While it was agreed that successful mechanisms for young people to access peer-to-peer support currently existed, increasing the availability and awareness of peer-to-peer support for all young people after treatment ends would be helpful, particularly for those in designated hospitals.

While healthcare professionals are reluctant to ‘hand hold’ after treatment ends, young people would find reassurance and comfort in knowing their healthcare team are still thinking of them. As previously identified, young people feel forgotten about when they complete treatment [15, 19, 20], therefore the recommendation of a standardised process for continued follow-up of young people’s psychosocial needs would facilitate healthcare teams to provide continued support and opportunities for signposting to other charities and services. An important challenge faced by young people was not knowing who to contact after treatment ended should they need support or information. Previous research has shown that many young adults want to access mental health support services after treatment ends but report not having access to these services or knowing how to access them [21].

Young people need to be motivated and self-aware to seek support in a new place, and lack of initiative to do this was described as a barrier to receiving support at the end of treatment. The assessment and identification of young people’s psychosocial needs should not happen by ‘chance’ but should be a routine aspect of a young person’s follow-up after treatment ends. More widespread, consistent, fluid and continued use of standardised holistic needs assessments after treatment is finished would enable ongoing social and emotional challenges young people face to be identified in a timelier way. This was advocated by NHS England in adult cancer services as part of the ‘recovery package’ [22] however the findings of this study illustrate that this is not routine practice in AYA cancer care. Routinely undertaking holistic needs assessments together with young people would enable and empower them to self-assess and identify areas where they may be struggling psychosocially. While the timing and structure of psychosocial follow-up and support provision has previously been identified as a problem [6], standardising this requires further research if we are to understand who would carry this out, and what this would look like for specific patient groups, such as those under disease surveillance, or those not in specialist AYA care settings.

The recognition of an individual’s influencing factors highlights an important direction for future research and practice. To better meet young people’s psychosocial support and information needs, support and information provided needs to be tailored to the individual’s personal circumstance, cancer type and age. Additionally, there needs to be greater recognition of the support needs of young people who do not experience an end of active treatment, such as those who have ongoing surveillance for their disease, but this still requires an adaptation to a new identity of living with and beyond their cancer.

The study identified specific groups of young people who were less likely to engage with services and events when treatment finished. This has not been shown previously and while it must be acknowledged that a limitation of this study was small numbers, not being ethnically diverse, nor was the full geographical spread of the UK being represented, this is something that warrants further investigation. Future research should explore the application of our model of adaptation to the end of treatment transition in a bigger and more diverse population.

Other limitations are that while the survey was distributed widely, we only had responses from 49 healthcare professionals. These represented the range of professionals attending the AYA MDT, and informal discussions with healthcare professionals attending a national conference where it was distributed indicated that some were completing on behalf of their organisation rather than individually. As an anonymous survey we did not ask the organisation to be named; however, the range of type of organisation and region represented suggests the survey captured the national organisational perspective if not the individual. We aimed to include 30 young people to interview and despite the variety of routes to recruitment we only managed to interview 11 young people. However, these interviews provided rich and detailed description of young people’s experiences when active treatment ended and included young people with a variety of cancer types who received their care in the range of care settings. Finally, the co-design workshop did not include medical representation. It would have been a useful addition to have the input of this perspective in the discussion. Nonetheless, we interviewed 12 healthcare professionals across different professional groups, and a wide range participated in the survey, therefore the perspective of the medical doctors is represented in the data as a whole. Despite these limitations, our results make an important contribution to understanding the needs of young people when active treatment ends and how these needs could be addressed.

## Conclusion

Young people are under prepared for the unpredictable and ongoing nature of both the physical and psychosocial issues they face at the end of their cancer treatment. Young people need timely, structured and equitable information and support provision at the end of treatment to prepare them for both the unexpected psychosocial issues that they may face, including their feelings of isolation and uncertainty about the future. We could further improve young people’s ability to adapt to life after treatment ends through structuring and standardising our approach to follow-up care, with a holistic focus that extends much further beyond the obvious physical concerns that young people present with during this transition. Importantly, this requires further work to establish a clearer alignment in relation to perceptions of end of treatment and transition. More research needs to explore the changes that can be made to the current processes, systems and environments in which young people are followed-up, and the content and availability of the resources they are signposted to, to assist young people’s adaptation to a ‘new normal’ at this time.

## Supplementary information

**Additional file 1.** Your individual thoughts and ranking about the most important areas for improvement for young people nearing the end of active cancer treatment.

**Additional file 2: Table S1**. Details regarding how services could prepare young people earlier around the ongoing impact of cancer and cancer treatment. **Table S2**. How standardised and continued follow-up of young people’s emotional well-being could be implemented. **Table S3**. Details of what information and resources specific to young people need to be developed, and the format of these resources.

## Data Availability

The data that support the findings of this study are available from Teenage Cancer Trust, but restrictions apply to the availability of these data, which were used under license for the current study, and so are not publicly available. Data are however available from the authors upon request and with permission of Teenage Cancer Trust.

## Abbreviations

AYA: Adolescent and young adult
CNS: Clinical nurse specialist
HNA: Holistic needs assessment
IAM: Integrated Assessment Mapping
MDT: Multidisciplinary team
NHS: National Health Service
TYAC: Teenage and Young Adult with Cancer (professional organisation)
UK: United Kingdom
YAP: Young Advisory Panel
YSC: Youth support coordinator
