# Supplementary material for: The support and information needs of adolescents and young adults with cancer when active treatment ends
Source: BMC Cancer. 2020 Jul 28;20:697. doi: 10.1186/s12885-020-07197-2 (PMC7388472; doi:10.1186/s12885-020-07197-2)
Supplement: Supplementary file 1 — Additional file 1. Your individual thoughts and ranking about the most important areas for improvement for young people nearing the end of active cancer treatment. [file 12885_2020_7197_MOESM1_ESM.docx]

**ADDITIONAL FILE 1**

**Your individual thoughts and ranking about the most important areas for improvement for young people nearing the end of active cancer treatment**

We would like you to spend some time before the workshop reviewing the seven ideas for service change or improvement for young people nearing the end of active cancer treatment.

These ideas have come directly from interviews with young people and healthcare professionals, a survey completed by healthcare professionals and current evidence published in the literature.

Please rank these suggestions from 1 – 7, 1 being the most important in your opinion and 7 being the least important in your opinion. Please write down any comments you have about why you have given that position, why you think it may make the most difference or the least difference.

If you have a suggestion of a key change or improvement that is not on the list, the blank row at the bottom of the table is for you to add your own suggestion (please rank it too).

We would like to request for you to keep these documents confidential at the moment.

PLEASE BRING THIS WORKSHEET WITH YOU TO THE WORKSHOP.

| **SUGGESTION TO BE RANKED** | **ADDITIONAL DETAILS ABOUT THE SUGGESTION** | **RANK POSITION**  **(1-7)** | **REASON** |
| --- | --- | --- | --- |
| Increasing the availability and awareness of peer-to-peer support for all young people after treatment ends | - Creation of peer support networks - Support and ideas from people who have gone through a similar thing - Have a Facebook or Instagram where social events and activities are posted - Access to either a support group or a buddy, someone who understands and that can share experiences with - Hospital-based support for young people of different ages, including those aged 25+ - More events/opportunities to meet people who have had cancer too to talk about it (in a non-hospital environment), share experiences - An online community for young people to connect after treatment |  |  |
| Earlier information provision and preparation around the ongoing physical and emotional impact of cancer and treatment after treatment ends | - Realistic expectations for young people about how they may feel at the end of treatment and how long it may take for them to physical and emotionally return to ‘normal’ - Better preparation at the beginning of treatment for what was going to happen and for the ongoing side effects of treatment, in a face-to-face conversation) - Having a jargon-free conversation with professionals to go through everything that happened just before ending treatment, or as treatment ended, including the long-term side effects to ensure the young person understands - Having a graded approach to assist young people to transition from on treatment to off treatment - More information provided about the end of treatment before treatment ends |  |  |
| Development of more information and resources specific to young people’s needs after treatment ends | - Access to more information/resources about emotional and physical health after cancer treatment ends - Existing information/resources made more available – pooled in one place - Health and wellbeing events/evenings specifically tailored to young people - Improved signposting of information - Information about ‘normal life’ that is not medically related, i.e. how to get back into dating after cancer treatment ends - Training healthcare professionals in supporting young people who are grieving for a life they could have had - Bereavement support for young people at the end of treatment who lose friends |  |  |
| Increasing awareness of support available to young people at the end of treatment | - Young people told what they have access to – that information made clearly available to them (rather than discovering what is available themselves) - Nurses/teams signpost young people to services they can access for emotional wellbeing, and they send reminders of these - Information about ongoing financial burden (time off work, costs of travel to and from hospital) and support with completing application forms for financial help after treatment ends - Be given a booklet to look at in your own time with information about ending treatment - A session where healthcare professionals talk through all of the things that are available and explaining why all of those things exist , including websites and forums - Gentle reminders from professionals when you come in for follow-up appointments about what support is available for young people to access - Access to good counselling services to help you deal with all of the emotions that come with finishing treatment - A healthcare professional who you trust explains what support is available for you to access, and encourages you to go |  |  |
| Improved communication and care co-ordination between all professionals involved in a young person’s care after treatment ends | - Assist young people to re-establish their relationship with their GP - Healthcare professionals across all settings of patient’s treatment pathways being informed of where young people are on their cancer timeline and when their treatment ends - Being informed of where young people are (geographically) when their treatment ends - Co-ordination of care to combine end of treatment support with follow-up clinics with Consultants |  |  |
| Clearer structures, roles and processes in place to assist young people to access support after treatment ends (e.g. definition of who is responsible for giving information and support at the end of treatment and how this is shared with the young person) | - In-house service reviews to understand how young people are followed up at the end of treatment - Having a navigator to assist young people to access support across whole pathway and beyond treatment - Provide young people with a complete list of names, roles and contact details of all professionals that were involved in their care, ideally provided by email - Provision of a clear protocol at the end of treatment so if a young person falls unwell suddenly they know what to do - Provision of a specific end of treatment CNS role - Specific end of treatment clinic - More formal use of end of treatment summaries - Clinical nurse specialists providing a specific end of treatment support service |  |  |
| Standardised and continued follow-up of young people’s emotional well-being after treatment ends | - More formal/consistent use of Holistic Needs Assessments (HNA’s) *(these are assessments done where healthcare professionals use a guide or checklist to assess the physical, emotional, practical, financial and spiritual well-being of patients. Patients may be offered an HNA at diagnosis, during treatment or after treatment has ended.)* - Consistency in follow-up - Standardised/formalised process of following patients at the end of treatment - A follow-up telephone call soon after ending treatment for reassurance, to see how the young person is doing, and direct them towards anything should they need it - Initial follow-up contact should be made after the last treatment: 4 weeks is too long to wait - More recognition from professionals that the ‘bubble’ of treatment has popped and that at end of treatment young people might feel “alone”/ not supported, without that protection safety net and regular hospital visits - Unprompted follow-up texts in between clinic appointments from professionals who know you and your treatment - Someone that knows you, through your whole treatment, and continues to follow you through at the end of treatment - Closer mental health follow up after treatment ends |  |  |
| Any other suggestions: |  |  |  |
