# Supplementary material for: The support and information needs of adolescents and young adults with cancer when active treatment ends
Source: BMC Cancer. 2020 Jul 28;20:697. doi: 10.1186/s12885-020-07197-2 (PMC7388472; doi:10.1186/s12885-020-07197-2)
Supplement: Supplementary file 2 — Additional file 2: Table S1. Details regarding how services could prepare young people earlier around the ongoing impact of cancer and cancer treatment. Table S2. How standardised and continued follow-up of young people’s emotional well-being could be implemented. Table S3. Details of what information and resources specific to young people need to be developed, and the format of these resources. [file 12885_2020_7197_MOESM2_ESM.docx]

**ADDITIONAL FILE 2**

**Table S1. Details regarding how services could prepare young people earlier around the ongoing impact of cancer and cancer treatment**

| Who | *“Best-placed person”* within the MDT, they may be based in the community. |
| --- | --- |
| When | ‘Drip-feeding’ information about the end of treatment from diagnosis onwards, but when and how fast this happens should be led by the patient. |
| Format | - Education for healthcare professionals, especially those not in specialist TYA care settings, about how and when to have these conversations to help to prepare young people for an uncertain future:   *"Maybe what we're truly managing is the uncertainty of future. So, maybe one of the 'Hows' is actually learning to increase their tolerance for how uncertain the future is going to be."* (Healthcare professional)   - Holistic needs assessments can be used as a structure for these conversations. - Use technology to increase/improve network-wide communication between healthcare professionals about the conversations that have happened. - Having a community-based support team to assist in providing information and preparing young people, thus also preparing young people for a transition away from the security of frequent hospital care. - Raising awareness and mindfulness around the impact of the language used in conversations with young people about their end of treatment experience, such as ‘easy’ or ‘short’ treatment and ‘getting back to normal quickly’. |
| Considerations | - This must involve setting realistic expectations with young people, providing honest and correct information, without young people losing hope or positivity. - It is therefore important that the language used in these conversations is carefully considered. |

MDT: multi-disciplinary team; TYA: teenage and young adult

**Table S2. How standardised and continued follow-up of young people’s emotional well-being could be implemented**

| Who | The young person’s keyworker, which is likely to be their CNS. Alternatively, the *“best placed person”* in the MDT, which may be the YSC or social worker. |
| --- | --- |
| When | It should be mandated that all young people have a Holistic Needs Assessment and are discussed formally in MDT at the end of their active treatment.  Young people considered one month as too long to wait to be contacted after their last day/session of treatment.  A framework for the keyworker/best placed person to guide and track their contact with young people at the end of treatment, to assist them to follow-up their emotional well-being in a structured way.  Timings within this framework should be patient-led; however, it should be standard that every young person is contacted within one month after treatment ends. |
| Format | Standardised framework to guide contact between keyworker/best placed person and young person when treatment ends.  This contact would be via young people’s preferred method of contact, most likely phone calls and/or texts.  Raising the profile and standardising end of treatment summaries, thus making more use of them to assist the provision of information at the end of treatment and to facilitate a conversation about potential ongoing issues as a result of a young person’s cancer and treatment. |
| Considerations | It is essential that there is a balance between *standardised* and *young people-led* follow-up: while standardising processes will increase equality, services need to remain as individualised and patient-centred as possible.  The differences in follow-up in paediatrics versus adult services need to be recognised. For young people, there needs to be standardisation, to attempt to align these two different models of follow-up:  *“What's interesting in this whole conversation is the disparity between follow up models in cancer. So again, looking at paediatrics and adult cancer follow up, it’s incredibly different. Again, if we’re thinking about, ‘Oh, we’re putting some recommendations out here,’ well, actually do we need to try and say, ‘We need to align those differences closer?’ ”* |

CNS: clinical nurse specialist; MDT: multi-disciplinary team; TYA: teenage and young adult; YSC: youth support coordinator

**Table S3. Details of what information and resources specific to young people need to be developed, and the format of these resources**

| Topics | - Bespoke information related to a young person’s individual treatment: details of the treatment they had; their side effects/potential side effects; and why they might experience these side effects. - Information related to being a young person, including: sex education and sexual health, relationships, education and career guidance, goal setting, motivation, life skills, mortgages, pregnancy. |
| --- | --- |
| Format | - Multi-media: ‘app’, email, audio recordings, online, booklets, more information in end of treatment summaries, an ‘exit pack’, a helpline, a ‘Teenage Cancer Trust guide to the end of treatment’ (available as online and paper versions). - Young people feel that letters by post are not very helpful. - Language needs to be age-appropriate and jargon-free. - Community spaces can be used as well to provide information and signpost resources. |
| Considerations | - An ‘app’ format would give young people back some responsibility and control over their knowledge and care. - Quality assurance of any information in a resource is essential to ensure it is trustworthy and credible. - It is important that young people are viewed as young people, regardless of their cancer, and therefore a large amount of the information and support they need is about the process of growing up, whilst going through a significant life event. |
